# Multi-Incidence Holographic Profilometry for Large Gradient Surfaces with Sub-Micron Focusing Accuracy

**DOI:** 10.3390/s22010214

**Published:** 2021-12-29

**Authors:** Moncy Sajeev Idicula, Tomasz Kozacki, Michal Józwik, Patryk Mitura, Juan Martinez-Carranza, Hyon-Gon Choo

**Affiliations:** 1 Faculty of Mechatronics, Warsaw University of Technology, 8 Sw. A. Boboli Street, 02-525 Warsaw, Poland; moncy.idicula.dokt@pw.edu.pl (M.S.I.); michal.jozwik@pw.edu.pl (M.J.); patryk.mitura.stud@pw.edu.pl (P.M.); juan.carranza@pw.edu.pl (J.M.-C.); 2 Media Research Department, Electronics and Telecommunications Research Institute, 218 Gajeong-ro, Yuseong-gu, Daejeon 34129, Korea; hyongonchoo@etri.re.kr

**Keywords:** surface reconstruction, longitudinal scanning function, autofocusing, angular spectrum, Fourier space, multi-incidence digital holographic profilometry, tilted thin element approximation

## Abstract

Surface reconstruction for micro-samples with large discontinuities using digital holography is a challenge. To overcome this problem, multi-incidence digital holographic profilometry (MIDHP) has been proposed. MIDHP relies on the numerical generation of the longitudinal scanning function (LSF) for reconstructing the topography of the sample with large depth and high axial resolution. Nevertheless, the method is unable to reconstruct surfaces with large gradients due to the need of: (i) high precision focusing that manual adjustment cannot fulfill and (ii) preserving the functionality of the LSF that requires capturing and processing many digital holograms. In this work, we propose a novel MIDHP method to solve these limitations. First, an autofocusing algorithm based on the comparison of shapes obtained by the LSF and the thin tilted element approximation is proposed. It is proven that this autofocusing algorithm is capable to deliver in-focus plane localization with submicron resolution. Second, we propose that wavefield summation for the generation of the LSF is carried out in Fourier space. It is shown that this scheme enables a significant reduction of arithmetic operations and can minimize the number of Fourier transforms needed. Hence, a fast generation of the LSF is possible without compromising its accuracy. The functionality of MIDHP for measuring surfaces with large gradients is supported by numerical and experimental results.

## 1. Introduction

Digital holographic microscopy (DHM) allows contactless reconstruction of the topography of an object from a single digital hologram [1]. The complex fields are extracted from the hologram, and then the phase is numerically unwrapped and converted into the corresponding topographic distribution, which is typically carried out by the thin element approximation method [2]. However, the difficulty of measuring the topography of reflective objects with discontinuities higher than λ/4 in conventional DHM has created the necessity of studying new approaches for solving this problem. These approaches are generally based on the use of spatial or temporal frequency diversity of the illumination. For example, the multi-wavelength interferometry (MWI) approach is a well-known solution for measuring the topography of objects with discontinuities [3,4]. This approach employs holograms that have been acquired using different wavelengths of illumination. Complex fields are recovered and numerical subtraction of two reconstructed phase maps allows generating a phase map with artificial wavelength, which has a significantly larger unambiguous measurement range (UMR). However, the MWI approach suffers from drawbacks, e.g., a tunable laser with a low tuning range, wavelength-dependent dispersion, and inhomogeneous absorption from the object [5]. Moreover, the optical system is prone to chromatic aberrations due to the employment of different wavelengths [6], and phase noise can be amplified due to the phase map subtraction [7]. 

Alternative approaches can be found in techniques that employ monochromatic light sources and illuminate the sample from multiple directions [8,9,10]. For example, optical contouring (OC) can perform a full-field surface measurement by using two holograms captured with different illumination angles [8,11,12]. OC can deliver UMR of up to few-millimeters, but unwrapping techniques might be required to obtain the absolute phase map. With the aim to get rid of the unwrapping process in surface reconstruction, multi-angle interferometry (MAI) was proposed [9]. MAI reconstructs the surface using more than two holograms. MAI increases the angular span of employed illumination angles (Δθ ϵ (0.5–1°)) and does not require unwrapping since the height information of each point is obtained independently [9]. Notably, OC and MAI offer surface reconstruction with large UMR, without the disadvantages of MWI, and with a simpler optical setup. Nevertheless, these techniques lack high axial resolution needed for inspection of objects with a few micrometers of height. 

The limitations found in OC and MAI have been addressed and solved by the multi-incident digital holographic profilometry (MIDHP) [10,13]. MIDHP assembles the set of object fields from the captured holograms for numerical generation of the longitudinal scanning function (LSF) [5,10,14,15,16,17,18]. The topography of the object is recovered by finding the maxima of LSF along the optical axis, which occurs when object fields are in focus. This is realized by sequential numerical propagation of the extracted complex fields for refocusing the optical wave-fields and determining the shape of the LSF at any given plane along the optical axis. Notably, the use of numerical propagation allows scanning of the object without mechanical displacement. Moreover, MIDHP can use different illumination strategies exploiting the full numerical aperture (NA) of the optical system. For example, reference [10] investigated the illumination strategies for MIDHP with a focus on the extension of the UMR. Thus, it was sufficient to illuminate the object from one side, which means that the employed angles illumination beams were all positive. Reference [13] made effort to minimize the illumination wave components to obtain a single shot multiplexing solution. 

The underlying theory of MIDHP was based on planar surface objects [10,13]. Thus, measured samples were intentionally selected as step-like objects (a set of flat surfaces with different heights). A key point for accurate topography reconstruction in MIDHP is the matching of phases of all propagated object waves. They will match when a single focus point is known with sufficient accuracy. Focus error introduces unproper reconstruction of the object shape. For the step-like objects, which do not require high focusing accuracy, manual focusing was sufficient to generate the LSF and complete measurement [10,13]. However, for objects with a continuous and high gradient shape, the accuracy of manual focusing is not sufficient. This is the main limitation of MIDHP when measuring more demanding samples. To solve this problem, there is a need for high accuracy autofocusing algorithms. Autofocusing algorithms can be carried out from a single hologram. The complex wavefield information is extracted and repropagated until a position of the plane with minimum sharpness in the reconstructed amplitude distribution is reached [19]. However, these techniques do not satisfy the accuracy criterion for the reflective phase samples. The reference [20] showed that phase-like samples are especially demanding for autofocusing algorithms and the focusing accuracy of image processing-based solutions is too low. Thus, a combination of coherent digital holographic microscope and low coherence interferometer was proposed to obtain high focusing accuracy [20]. However, such a system is experimentally complex and time-consuming since the method requires mechanical movements and parallel processing of data from two different systems.

In this work, a novel MIDHP is presented for topography reconstruction of objects with a high gradient of a continuous surface. To preserve the functionality of the LSF for object areas of high tilts, the illumination scanning is carried out on two symmetrical sides and includes more illumination components. Linear and geometrical illumination scanning strategies are analyzed with numerical experiments. The proposed high accuracy autofocusing algorithm is based on a comparison of shapes obtained by the LSF and the Thin Tilted Element Approximation (TTEA) [21]. The calculated focus measure is evaluated for a selected region of the continuous shape. It is proven with numerical and optical experiments that the proposed autofocusing algorithm is capable to deliver in-focus plane localization with the required submicron resolution. 

The computational load of MIDHP, when testing samples with high gradient, substantially increases compared to previous implementations that reconstruct discontinuous objects. This is due to (i) scanning with a larger number of illumination components, and (ii) the application of the proposed autofocusing algorithm. The classical generation of the LSF is based on the angular spectrum method [22], where the summation of the propagated wave is realized in the spatial domain [10,13]. Consequently, the LSF generation algorithm with *P* illumination scanning waves needs evaluation of 2P Fourier Transforms (FTs) for one propagation step. To reduce the computational load, in this work we propose an LSF generation algorithm with a summation scheme realized in Fourier Space. This allows developing an algorithm that needs the evaluation of only one FT for one propagation step. The computational load of the LSF generation algorithm is further reduced by employing the zero-padding technique where frequency summation is realized at a decreased number of samples. This is possible because in the frequency domain the components of the propagated fields occupy a limited space. Sequentially after the summation operation, there is frequency zero-padding, thus the LSF of the original sampling rate is obtained. As a result, faster surface reconstruction is possible without compromising the accuracy of MIDHP. It is worth noting that the reference [17] stated that shape reconstruction of tilted surfaces when using the LSF had a systematic error, which depends on the distance between the reference mirror and the plane of focus of the imaging system. The magnitude of the error also depends on the slope angle of the tilted plane and the illuminating aperture. However, the MIDHP approach has proved that such systematic error is not presented anymore. The principle of work of this paper is demonstrated by using numerical and optical experiments. 

MIDHP is composed of hardware and system solutions and the combination of these solutions makes a robust technique for reconstructing any kind of surface. This paper is organized as follows. Section 2 describes the experimental system. Section 3 introduces a numerical algorithm for fast LSF evaluation. Section 4 presents the strategies for object illumination, and numerically analyses the metrological functionality for high gradient objects. Section 5 explains the auto-focusing algorithm developed in this work. Section 6 carries out the experimental reconstruction of the sample. Finally, Section 7 gives the corresponding conclusions.

## 2. Measurement System

Our research involves the application of different numerical methods for processing of precisely collected measurement data. Measurement process of the MIDHP method requires an adaptation of the digital holographic microscopy system with normal illumination direction to the setup with a possibility of controlled change of illumination direction with large angular range. This section describes the experimental system and measurement object.

**Figure 1:**
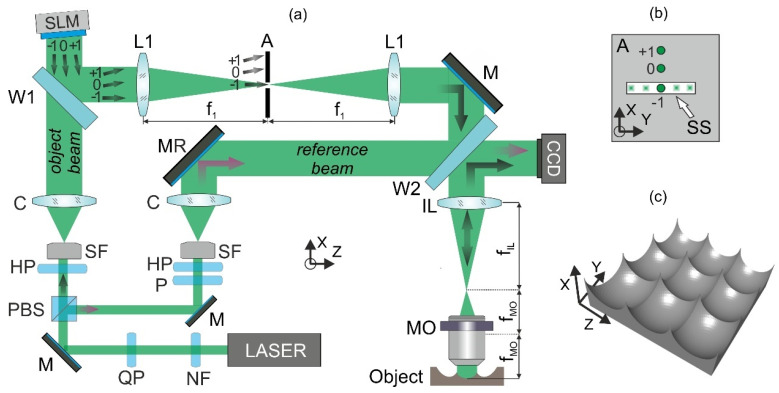
(**a**) Scheme of the measurement system, (**b**) details of the aperture A (view from illumination direction), (**c**) visualization of the test object.

To verify MIDHP features experimentally, we have built the measurement system based on the Twyman-Green interferometer (Figure 1a). The coherent light source is Nd-YAG laser (λ = 532 nm). The laser beam is attenuated by a neutral density filter NF and is elliptically polarized with quarter-wavelength plate QP. Then the laser beam is divided by a polarization beam splitter cube PBS in two orthogonally, linearly polarized beams. The beams are spatially filtered by SF, collimated by lenses C, and directed into the interferometer arms. The linear polarization of the object beam is adjusted by a half-wavelength plate HP according to the requirements of the employed spatial light modulator (SLM). The beam reflected from the SLM is directed by an optical wedge W1 to the afocal optical system consisting of two identical lenses L1 (f_1_ = 125 mm). This beam, after reflection from mirror M, passes through a second afocal system including an imaging lens IL (f_IL_ = 200 mm) and a microscope objective MO (Mitutoyo Infinity-Corrected Long Working Distance Objective, 50×, *NA* 0.75). Distance from L1 to IL is equal to f_1_ + f_IL_. The beam exiting MO illuminates the measured surface. The reflected object beam goes back through the afocal imaging system and is directed by optical wedge W2 towards the CCD camera (Basler Pilot, resolution 2456 × 2058, pixel size 3.45 μm). The CCD sensor is set at the distance f_IL_, which realizes optical conjugation between object and image plane (CCD). The path of the second beam, consisting of a reference of an interferometer, is shorter and much less complicated. The flat wavefront is reflected by a mirror MR directly to the CCD camera. The azimuth of the linear polarization of the reference beam is adjusted by the HP while the polarizer P limits its power. Object and reference beams are combined by the optical wedge W2. The polarization elements allow matching polarization of beams and obtain optimal contrast of the resulting interference pattern. The tilt of the MR is used to introduce a high spatial frequency of interference fringes required to separate the object beam from the zero-order and its conjugate.

Figure 1c shows a visualization of the micromold array. The measurement object in our experiment is a single micromold from an array of elements etched into silicon substrate [23]. The micromold shape is a square (edge length of 194.5 µm) with a smooth, continuous and reflective surface of maximum depth up to 12 µm. The object features (shape complexity, depth and surface gradient) are a challenge for MIDHP, and the shape reconstruction cannot be obtained using known approaches [10,13]. In this work, we propose a novel MIDHP enabling such a challenging measurement. The reconstruction of the object topography will be carried out by assembling a set of object fields from holograms captured using a different illumination direction. Therefore, the key element of the system with different illumination directions is a spatial light modulator SLM (HOLOEYE Photonics AG, model GAEA-2, reflective phase only liquid crystal on silicon microdisplay, resolution 4160 × 2464, 3.74 µm pixel size). The incoming beam is split into diffraction orders, according to the phase map in the SLM. Only −1st diffraction order is selected at the Fourier plane of lens L1 by opening a rectangular aperture A (Figure 1b). The aperture’s role is to block all unwanted orders during the whole process of scanning. An additional tilt of the SLM enables to shift away from the undesired 0th diffraction order and direct the −1st order on an optical axis of the system. In this situation, the object is illuminated in the normal direction. Unfortunately, due to back reflections obtained from internal surfaces of the MO in the system, information from illumination angles within the range (−5°, 4.5°) are perturbated with parasitic fringes and not useful in the reconstruction procedure. The change of illumination angle is realized by modification of the phase map at the SLM. The vertical direction of scanning is precisely adjusted by an area of scanning slit (SS) at the aperture plane. The vertical aperture and scanning enable filtering higher diffraction orders, which are generated by the SLM in the horizontal direction. Using larger tilts of illumination, an object beam with information about higher surface slopes can be captured, also measurements with higher axial resolution can be realized [10]. The accuracy of the illumination angles was evaluated experimentally with a flat mirror as an object. Within the scanning range from −30° to 30° the maximum error does not exceed 0.1°. Thus, the proposed measurement system delivers extended information about an object in a short time without any mechanical movements. 

## 3. Improved Algorithm for Fast LSF Evaluation

The algorithm for recovering the 3D shape of an object in the MIDHP method is based on the evaluation of the LSF, which uses numerical wave propagation with the angular spectrum (AS) method [22]. Calculation of AS is a major component of the numerical computation of the LSF. For a given depth of the object, the corresponding LSF is determined with two fast Fourier transform (FFT) operations for each direction of object illumination. To evaluate the LSF, obtained results with the AS method are summed in the spatial domain. In this work, we propose the LSF generation method with a summation scheme carried out in Fourier space, which enables minimization of the number of FFTs needed and consequently improves the evaluation speed of the LSF. 

The AS based algorithm for evaluation of the LSF of Ref. [13] can be presented by:

(1) $$LSF(x,z)=\left|\sum_{p}\int \int \tilde{u}p(f)H(f,z)\exp\{2\pi ixf\}df\exp\{2\pi i(xf_{p}+zf_{zp})\}\right|$$

where $x=[x,y],f=[f_{x},f_{y}]$, $\tilde{u}p$ is the FT of the reconstructed object wave, $H(f,z)=\exp\{ikz{\left(1-\lambda^{2}f^{2}\right)}^{1/2}\}$ is the AS phase accommodation kernel, the subscript p∈(1:P) indicates the direction of the illumination wave vector ***k^p^*** and consequently, $f_{p}=[f_{px},f_{py}]$ is the spatial frequency of illumination plane wave, *f_zp_* is the corresponding longitudinal frequency, and *p* is the number of illumination waves. By using shifted frequency coordinates $f+f_{p}$ and after analytical manipulations in the algorithm, Equation (1) can be rewritten in the form:

(2) $$LSF(x,z)=\left|\int \sum_{p}{\tilde{u}}_{cp}(f)H(f+f_{p},z)\exp\{2\pi izf_{zp}\}\exp\{2\pi ixf\}df\right|$$

where ${\tilde{u}}_{cp}(f)$ is the FT of the object wave without illumination carrier of *p* component:

(3) $${\tilde{u}}_{cp}(f)=\iint u_{p}(x)\exp\{2\pi ixf_{p}\}\exp\{-2\pi ixf\}dx$$

**Figure 2:**
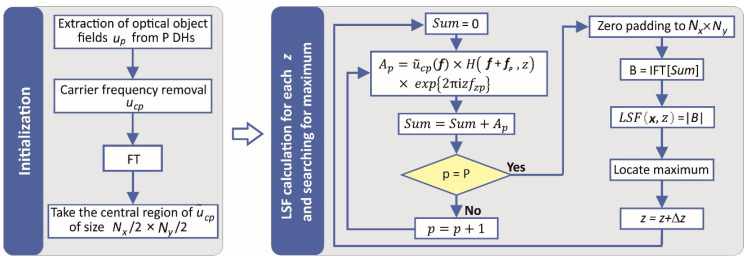
Diagram of numerical procedure for evaluation of LSF.

Figure 2 presents main steps of the implemented numerical procedure for evaluation of LSF maximum for each step *z*. In the initialization step, each of the input holograms is processed to obtain the object wave in the frequency domain. The central region is stored and used in the second step of numerical procedure of LSF evaluation. In the calculation scheme of Equation (2), the summation is realized in Fourier space. As a result of the proposed modification, for each step *z*, the algorithm needs only one FFT. For comparison, the algorithm based on Equation (1) requires computations of 2*P* FFTs per step. There is an additional advantage of the algorithm based on Equation (2). All of the operations are realized in the Fourier domain, and thus, unused high frequencies of the hologram (due to limited *NA* of optical elements in DHM) can be omitted in the algorithm. In our implementation, the summation part of the algorithm is only evaluated for the central region of the object waves spectrum (*N_x_N_y_*/4 discrete positions). After summation and before inverse FFT, zero padding is realized to have the full frequency range of the MIDHP system. In this way, there is no reduction in the sampling rate. This part of the algorithm is referred to as the padding part.

A speed test was performed to compare the efficiency of all three versions of the algorithm: based on Equation (1) (A1), based on Equation (2) without (A2) and with (A2P) the padding part. The calculations were performed for the parameters of our MIDHP, *p* = 28, scanning range 20 μm and the propagation step 0.05 μm. The speed of calculations is 1560.2 s, 388.4 s, 141.1 s for A1, A2 and A2P, respectively. Hence, in this work for all simulations and experiments, the algorithm A2P is employed, which is 11 times faster calculations. 

## 4. Illumination Strategies for Large Gradient Objects

**Figure 3:**
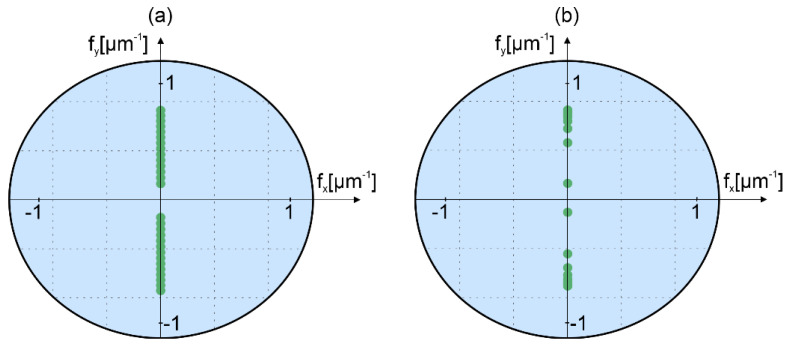
Frequency distribution of illumination beams in (**a**) equally angle spaced strategy; (**b**) geometrically frequency spaced strategy. The circle shows the *NA* limit of the imaging system.

Reference [10] investigated the illumination strategies for MIDHP with a focus on the extension of the measurement range. Thus, it was sufficient to illuminate the object from one side, where the corresponding angles were all positive. Meanwhile, reference [13] optimized frequency separation of the illumination wave components into *NA* of the imaging system. This was done to obtain a single shot hologram in which seven directions of illuminations are multiplexed. In both works, the measured samples were a composition of flat surfaces with different heights. Here, we focus on the measurement of objects with continuous high gradient shapes. For such objects, it is important to illuminate them from different directions. Thus, the LSF function will not lose its performance for areas of the object that has a high inclination of surface. Reference [10], which proposed the LSF, developed the theory for a plane object with a surface normal to the optical axis. For objects of complex shape, as studied in this paper, this theoretical solution is not applicable. Thus, we propose a numerical experiment, which verifies the metrological parameters of the LSF. In this section, two illumination strategies are introduced and numerically tested. The frequency distributions of the proposed illumination schemes are presented in Figure 3, where illuminations angles close to the optical axis are excluded. 

The first strategy, illustrated in Figure 3a, applies illumination angles in the y-direction, which are separated by a constant angle step; this strategy provides a large UMR and reduced measurement noise [10]. The angular range of illuminations is [−29°, −5°] for negative and [4.5°, 29.5°] for the positive side. The use of different ranges for both sides is associated with obtaining different values of the longitudinal vector components of the corresponding illumination beams. The total number of used angles is *p* = 28, and thus, for each side, we use fourteen illumination components with the angular step of *δθ* ≈ 1.85°. 

The second illumination strategy, shown in Figure 3b, employs geometrically frequency series [10], which is here calculated with progression factor *R* = 1.58. To determine this optimal value of *R*, heights of the side lobes were investigated numerically as in reference [13]. The geometrical strategy enables measurements with a reduced number of illumination directions. The illuminations angles are within the same range as for the first strategy. For the geometrical series, there are nine illuminations on negative and positive sides (*p* = 18). 

**Figure 4:**
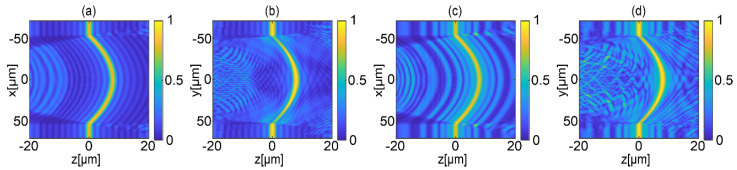
Numerical LSFs for a 3D micro focusing object when using different illumination scanning strategies: (**a**) horizontal and (**b**) vertical cross-section for equally angle spaced strategy, respectively; (**c**) horizontal and (**d**) vertical cross-section for geometrically frequency spaced strategy.

To test the performance of the proposed illumination strategies, the measurement process of high gradient objects in MIDHP shown in Figure 1 was simulated using the Born series expansion method [24]. As a sample, a 3D focusing object having a spherical shape with a maximum slope of 17.5° (maximum height of 8 μm) was used. The result of the simulation is *P* scattered object waves for both proposed illumination strategies. These waves are the basis of a numerical experiment for verifying the metrological parameters of the LSF. The LSF was calculated for the range [−20 μm, 20 μm] with step 0.005 μm. Figure 4 presents obtained LSFs in x-z and y-z planes for two illumination scanning approaches. The y-z plane is selected because illumination components are along the y axis. Moreover, for vertical cross-section, the selected object has zero gradients in direction-direction. Thus, the selected illumination strategies are best suited for the investigated object for the y-z plane. While conducting similar considerations, the x-z plane of objects is the least optimal case.

Figure 4a,b depicts the LSFs for equally angle spaced strategy, while Figure 4c,d is for geometrically frequency spaced strategy. The depicted LSFs show that the shape of the object can be recovered properly for investigated measurement range using both considered illumination scanning strategies. The LSFs for each transverse coordinate have distinct and well resolvable maximum, and, for the entire scanning range for both cross-sections, the side lobs have small values. For the equally angle spaced strategy, side peaks of the LSFs are smaller than 0.5 for the negative and positive values of the propagation distance. Regarding the second illumination approach, the LSFs take values smaller than 0.6. Despite larger side peaks in the geometrically frequency spaced strategy, its LSF has sufficient quality for the proper object shape recovery. 

**Figure 5:**
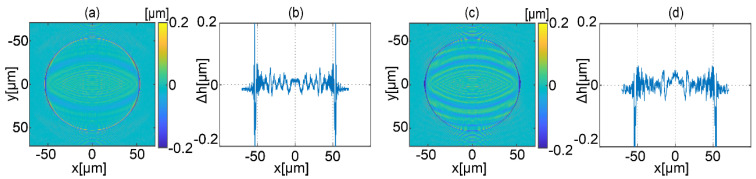
Comparison of the shapes of the simulated and reconstructed object: (**a**) shape difference map and (**b**) corresponding horizontal cross-section for equally angle spaced strategy, (**c**) shape difference map and (**d**) horizontal cross-section for geometrically frequency spaced strategy.

In Figure 5, comparison between simulated and reconstructed object shapes is presented. Figure 5a,c shows the shape difference map between simulated (ideal) and recovered object shapes for equally angle and geometrically frequency spaced strategy, respectively. Figure 5b,d shows the corresponding horizontal cross sections. For both strategies, the largest errors occur on the edges of the reconstructed object. The main reason is the large gradient of shape for the area connecting the flat surface with a curved one. Nevertheless, the object is reconstructed properly employing both strategies. Differences in shape are small. Excluding the shape transition area, peak to valley (PV) and standard deviation (STD) errors are, PV = 0.20 μm, STD = 0.020 μm and PV = 0.28 μm, STD = 0.027 μm for equally angle and geometrically frequency spaced strategies, respectively.

## 5. High Accuracy Autofocusing Method

**Figure 6:**
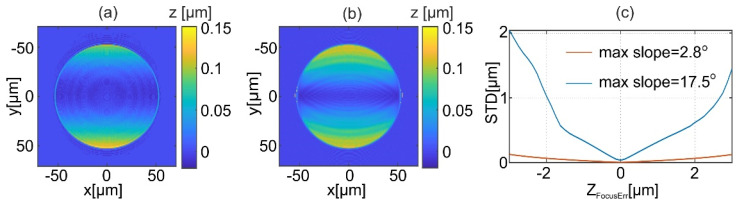
The difference of shapes obtained using LSF for defocus *z_d_* = −1 μm for object of maximum slope 2.8° (*NA* = 0.025) (**a**) and 17.5° (*NA* = 0.15) (**b**). The standard deviation of shape reconstruction error as a function of defocus error for two different objects (**c**). The calculations are performed for the geometrically frequency illumination scanning (Figure 3b).

The accuracy of the reconstructed shape relies on matching of the phases of all object waves captured in MIDHP in the measurement sequence. The 3D phase distributions match when a single focus point is known with sufficient accuracy. Focus error leads to improper shape reconstruction of the investigated object. To illustrate this effect, a simulation of the measurement process in MIDHP was performed with intentionally introduced defocus error *z*_d_ = −1 μm. Two micro focusing objects of spherical shape were simulated, first with low (maximum slope 2.8°, maximum height 1.3 μm) and second with high gradient (maximum slope 17.5°, maximum height 8 μm). Reconstruction errors are illustrated in Figure 6a,b for low and high *NA*, respectively. These error maps are obtained by subtraction of the defocused object reconstruction from the reconstruction for best focus. Illumination components of employed geometrically frequency illumination scanning are along the y-axis with angles shown in Figure 3b. Thus, for both cases, stronger errors are along this axis. Figure 6c presents the standard deviation (STD) of shape reconstruction error objects as a function of defocus error. It can be noticed that the reconstruction error is considerably larger for objects with higher *NA*. 

To minimize the shape reconstruction error due to the focusing inaccuracy, here an algorithm for finding the object focus with sub-micron accuracy is proposed. The algorithm calculates the focus measure based on the evaluation of shapes for selected area ***x*_Ω_**: = ***x***∈Ω. The proposed approach compares shape calculated using LSF and the TTEA [21]. The TTEA enables shape reconstruction for objects of continuous shape with low *NA* and large object illumination angle. It was shown that for objects having *NA* smaller than 0.05, the approximation provides accurate shape results [21]. For the parabolic focusing object of refractive index 1.5, the *NA* of 0.05 corresponds to the maximum object slope of 5.7° [25]. Thus, within the focusing algorithm using the TTEA, the shapes are calculated for all *P* illumination wave components and for the selected area having a continuous surface of low gradient. 

The proposed focus measure for defocus *z_d_* is given by:

(4) $$F(z_{d})=\sqrt{\sum_{p}STD(z_{rec}(x_{\Omega },z_{d})-z_{TTEA(p)}(x_{\Omega },z_{d}))}$$

where *z_rec_* is the shape reconstructed using the LSF, STD is standard deviation, while

(5) $$z_{TTEA(p)}(x_{\Omega }+x_{s},z_{d})=\frac{\Psi_{p}(x_{\Omega },z_{d})}{2k_{0}\cos(\theta_{p})}$$

is the shape reconstructed by the TTEA, *θ_p_* is the angle of wave vector of illumination wave, and Ψ*_p_* is unwrapped phase of *p* object wave. In this work, illumination angles are contained in y-z plane, thus the components of transverse vector ***x_S_*** are ***x****_S_* = 0 and $y_{S}=\tan(\theta_{p})z_{TTEA(p)}(y)$, respectively. 

The autofocusing algorithm consists of the following steps: (i) initialization, (ii) propagation of object wave fields for *z_d_*, (iii) setting phase to zero at focus point ***x_f_*** for all wave fields, (iv) calculation of focus measure F(*z_p_*), (v) finding minimum of focus measure within search range *Z_s_*, (vi) focus correction of object waves. In the initialization step focus point ***x*_f_**, focus area ***x*_Ω_**, and focus depth search range *Z_s_* are set. Steps (ii)–(iv) are executed for each considered depth *z_d_* ∈ *Z_s_*. In step (ii) all object wavefields are refocused, then the phase of all waves is adjusted to have zero phase at the focus point, next the Equations (4) and (5) are evaluated to find the focus measure. In step (v) minimum of focus measure is found and finally in (vi) the focus of all object waves is corrected for the evaluated focus depth.

**Figure 7:**
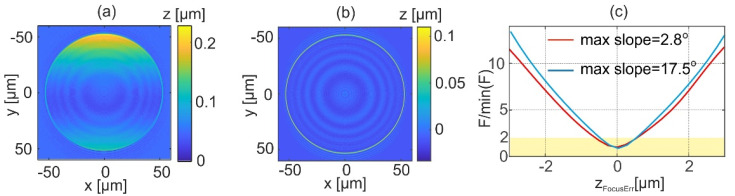
The difference of shapes obtained using LSF and TTEA for *θ*_p_ = −29° for defocus *z_d_* = −1 μm (**a**) and 0 μm (**b**). The focus measurement for two different objects (**c**); the calculations performed for the geometrical frequency illumination scanning (Figure 3b).

Figure 7 illustrates the effects of the proposed autofocusing algorithm on a reconstructed shape. For this purpose, the measurement of micro focusing object of maximum slope 2.8° was simulated for geometrical frequency illumination scanning (Figure 3b). Subsequently, the object was reconstructed for different defocuses (*z_d_* = −1, 0 [μm]). Figure 7a,b shows the difference between shapes reconstructed using LSF and TTEA for two defocus values. For TTEA the most off axis angle is selected for *p* = 1 (*θ*_p_ = −29°) is selected to visualize the focus errors obtained for one of the beams. It is showed that for proper focus (*z_d_* = 0 μm) the phase of all illuminations agrees and there is no difference between approaches taken for shape reconstruction. However, for the defocus error there is a significant difference between the results provided with different approaches. It is worth noting that for different illumination angles the obtained differences have similar distributions. For investigated defocus the difference is no larger than 100 nm. Final plots are presented in Figure 7c. It shows focus measures as a function of defocus error, which were calculated for two different objects (maximum slope 2.8° and 17.5°). The sizes of the area considered are 33 × 33 μm^2^ for the high *NA* object, and 69 × 69 μm^2^ for the low *NA*. Figure 7c shows normalized focus measures to the smallest obtained value. The simulations show that the proposed focus measure has a distinct minimum. The yellow rectangle is added to indicate a region where the normalized focus measure *F* grows to 2. It shows that this value corresponds to a focus error smaller than 1 μm. The usefulness of the minimum is investigated in the experimental Section 6. 

## 6. Experimental Results

This section presents experimental results of the micromold structure by two proposed illumination scanning methods. Both illumination scanning approaches are realized along the *y*-axis, as illustrated in Figure 3, within the range [−29°, −5°] for negative and [5.5°, 29.5°] for positive angles. For the equally angle spaced strategy *p* = 28 holograms in total were acquired, while for the geometrical series strategy *p* = 18 holograms were used. 

**Figure 8:**
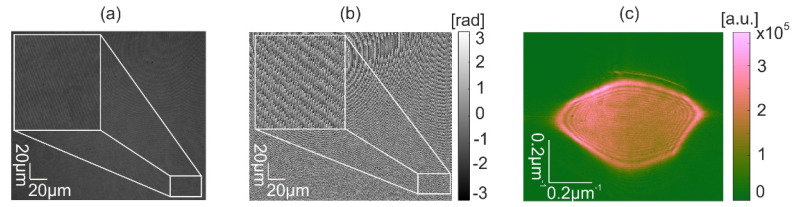
Example of experimental data: (**a**) recorded hologram, (**b**) phase, and (**c**) Fourier spectrum of the object in linear scale.

All holograms were captured with plane wave reference having carrier frequency, which is sufficiently large to shift the entire object wavefield from the zero and correlation orders. Thus, the full information of object wave can be recovered from a single holographic frame by a sequence of operations: (i) multiplication with the reference wave, (ii) low pass filtering to the *NA* of optics of MIDHP, and (iii) subtraction of system aberration [13]. Figure 8 illustrates data captured by one of the holograms. Figure 8a shows the intensity distribution of the hologram; Figure 8b depicts the phase of the reconstructed object beam; and finally, Figure 8c illustrates the object beam in the Fourier space. 

**Figure 9:**
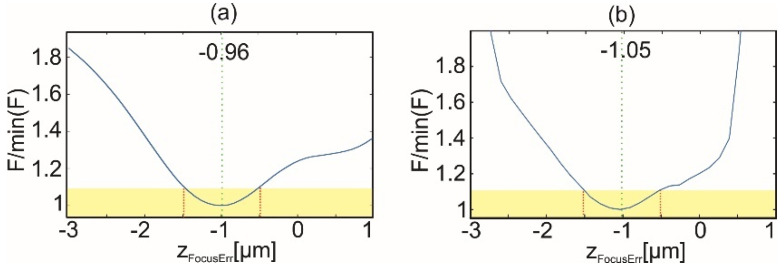
The focus measurement for: (**a**) equally angle spaced strategy, and (**b**) geometrically frequency spaced strategy.

Before recording the hologram data, the focus was set manually to the central region of the object (center of the hologram). The alignment of the object was based on observation of the reconstructed amplitude of the object wave. The autofocusing algorithm of Section 5 was applied for finding the focus point more accurately. The distribution of autofocusing algorithm for equally angle spaced illumination scanning is presented in Figure 9a. The focus curve was obtained applying step size 0.01 µm in axial scanning range [−3, 1] µm. The obtained focus curve has a clear minimum at −0.96 µm, indicating the axial shift required to reach focus at the selected point of focusing distance. A similar calculation of focus curve was performed for geometrically frequency spaced strategy. Figure 9b shows the focus measure for this set of experimental data. The focus measure curve has a minimum indicating the value of focusing distance equal to −1.05 µm. The obtained difference between the two focus distances is 100 nm indicating the submicron focusing accuracy of the proposed method. This difference is due to not fully compensating the system aberrations and hologram noises (speckle noise).

**Figure 10:**
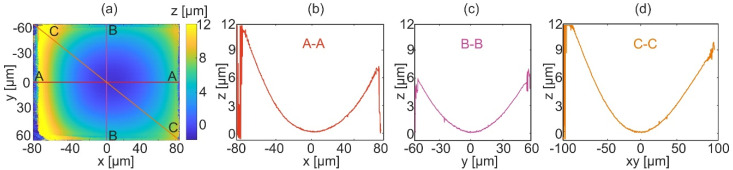
Results of the topography reconstruction of the micro mold with the equally angle spaced strategy: (**a**) 2D map, (**b**) horizontal cross-section (A-A), (**c**) vertical cross-section (B-B), and (**d**) diagonal cross-section (C-C).

The results of the topography reconstruction of the measured sample for equally angle spaced strategy are presented in Figure 10. Before calculation of the LSF, acquired and reconstructed object wavefronts were refocused by the focus value of −0.96 µm and then the procedure of topography reconstruction was completed. Figure 10a shows the result of the reconstruction of the micromold. The horizontal, vertical, and diagonal cross-sections are presented in Figure 10b–d, respectively. Those plots confirm a continuous reconstruction with low-level noise on the entire area of the measured object. 

**Figure 11:**
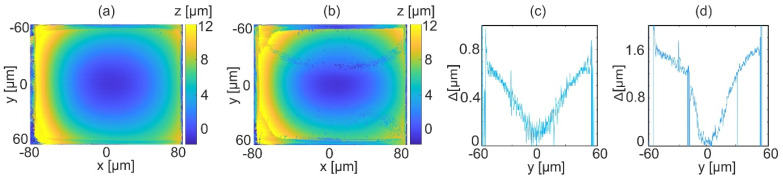
Influence of focusing distance on reconstructed shape: (**a**) topography reconstruction at z = −1.96 µm, (**b**) topography reconstruction at z = −3.96 µm, (**c**) difference in vertical cross-sections of the reconstructed object at z = −0.96 µm and −1.96 µm, and (**d**) difference in vertical cross-sections of the reconstructed object at −0.96 and −3.96 µm.

The usefulness of the proposed autofocusing method is analyzed by comparison of reconstructions for the best focus (calculated focusing distance −0.96 µm, results shown in Figure 10) and for focus error of 1 µm and 3 µm (focusing distance −1.96 µm and −3.96 µm). The reconstructions for intentional focus error are presented in Figure 11a,b. It can be observed that with the increase of focusing distance from the best focus, the quality of reconstruction decreases. This is seen by generated shape discontinuities and higher noise levels. The numerical experiment presented in Figure 7 shows that for small focus error continuous shape reconstruction error for scanning direction is obtained. Thus, Figure 11c,d presents vertical cross-sections at the center of difference between the reconstructed object for best focus and for focus error −1.96 µm, −3.96 µm, respectively. The obtained shape reconstruction errors are consistent with those predicted by the numerical experiment. Plots presented in Figure 11c, d shows resulting shape reconstruction errors up to 0.7 µm and 1.6 µm for focusing error 1 µm and 3 µm, respectively. Other phase-based autofocusing algorithms were tested as well. The NORM L1 [26] and VAR [27] algorithms were used to calculate the focus plane. However, the obtained focus distances were not correct leading to non-continuous and erroneous shape reconstructions. This type of phase-focusing algorithms is based on the criteria of minimum amplitude variations, which is not fulfilled by the continuous reflective objects. 

**Figure 12:**
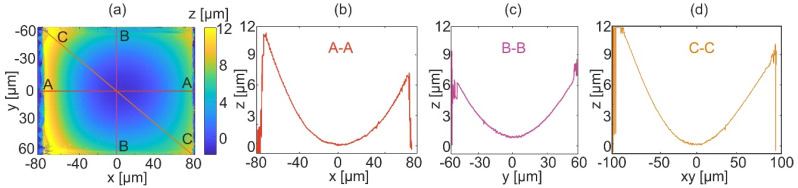
Results of the topography reconstruction of the mold with geometrically frequency spaced strategy: (**a**) 2D map, (**b**) horizontal cross-section (A-A), (**c**) vertical cross-section (B-B), and (**d**) diagonal cross-section (C-C).

For the second part of the experiment, eighteen holograms were captured with scanning along the *y*-axis using the geometrically frequency spaced strategy. The reconstructed object topography is shown in Figure 12. Figure 12a shows the full field reconstruction of the micromold shape. Horizontal, vertical, and diagonal cross-sections of the reconstructed shape are shown in Figure 12b–d. 

The result shows that using the geometrically frequency spaced scanning it is possible to obtain a reconstruction quality comparable to the equally angle spaced strategy. Comparison of the reconstructions shows difference only at noise level. Moreover, the depth of the micromold has the same value in both reconstruction procedures. The advantage of the second technique is the lower number of captured holograms, which reduces the overall measurement time (data registration and data processing time) by 35%, approximately.

## 7. Conclusions

In this article, we have shown that it is possible to overcome the limitations of the MIDHP for measuring objects with challenging features including continuous change of shape and high gradient. The implemented solution relies on high focusing accuracy and numerical generation of the LSF for reconstructing the object topography. To satisfy the need for high precision focusing, an autofocusing algorithm based on the comparison of shapes obtained by the LSF and the TTEA was proposed and tested. It is proven that this autofocusing algorithm is capable to deliver in-focus plane localization with submicron resolution. The reconstructed object wavefronts are refocused by a determined focus value and then the procedure of topography reconstruction based on LSF is carried out. The second achievement of reported work is the acceleration of calculations needed for the reconstruction of object topography. In our implementations, the LSF generation algorithm with summation scheme realized in Fourier space is proposed. This allows developing the algorithm that needs evaluation of only one FT for one propagation step. The computational load is further reduced by employing the frequency zero-padding technique. When comparing with previous MIDHP implementation, the amount of time for generating the LSF is reduced by a factor of 11. The proposed algorithm enables a fast generation of the LSF without compromising its accuracy. As an integral part of MIDHP, linear and geometrical illumination scanning strategies were analyzed. For an object of complex shape, MIDHP performance test with numerical experiment verifying meteorological parameters of the LSF was carried out. For equally angle spaced strategy, 28 holograms in total were acquired, while for geometrical series 18. 

The experimental reconstructions of a micromold structure prove that MIDHP can be successfully applied for measuring the topography of objects with a high gradient of a continuous surface. The results confirm a continuous reconstruction with small noise on the entire area of the measured object. Notably, the linear strategy provides a smoother surface reconstruction, but larger processing time is required while the geometrical series strategy reduces time but increases the amplitude of surface artifacts.

## Data Availability

Data underlying the results presented in this paper are publicly available at this time but may be obtained from the authors upon request.
